# Supplementary material for: Influenza Vaccine Effectiveness Against Pneumonia and COPD Exacerbations Among Patients with Chronic Obstructive Pulmonary Disease in Thailand: A National Test-Negative Design Study, 2013–2024
Source: Vaccines (Basel). 2026 Aug 18;14(8):711. doi: 10.3390/vaccines14080711 (PMC13517751; doi:10.3390/vaccines14080711)
Supplement: Supplementary file 1 [file vaccines-14-00711-s001.zip › vaccines-4464949-supplementary.pdf]

Table S1. Vaccine effectiveness by influenza activity level, Thailand, 2013–2024.

Influenza Activity Level	Outcome	Adjusted RR	95% CI (RR)	VE %	95% CI (VE)
Low	Pneumonia	0.441	0.358–0.543	55.9	45.7–64.2
	COPD exacerbations	0.343	0.234–0.502	65.7	49.8–76.6
Moderate	Pneumonia	0.440	0.370–0.522	56.0	47.8–63.0
	COPD exacerbations	0.323	0.200–0.522	67.7	47.8–80.0
High	Pneumonia	0.481	0.390–0.594	51.9	40.6–61.0
	COPD exacerbations	0.306	0.171–0.547	69.4	45.3–82.9

Abbreviations: CI, Confidence Interval; COPD, Chronic Obstructive Pulmonary Disease; RR, Risk Ratio; VE, Vaccine Effectiveness. **Notes:** VE was calculated as (1 − adjusted risk ratio) × 100. Estimates were obtained using modified Poisson regression with cluster-robust standard errors at the province level. Analyses were restricted to RT-PCR–tested episodes and conducted using a test-negative design framework. Models were adjusted for age group, sex, comorbidity status, smoking status, COPD severity, healthcare utilization, and influenza season.

Table S2. Southern Hemisphere influenza vaccine components and yearly distribution of circulating influenza virus types in Thailand for years with available descriptive strain-context information, 2011–2015.

Vaccine Strain Composition	2011	2012	2013	2014	2015
**A (H1N1)**	A/California/7/2009 (H1N1) pdm09-like virus	A/California/7/2009 (H1N1) pdm09-like virus	A/California/7/2009 (H1N1) pdm09-like virus	A/California/07/2009 (H1N1) pdm09	A/California/07/2009 (H1N1) pdm09
**A (H3N2)**	A/Perth/16/2009 (H3N2)-like virus	A/Perth/16/2009 (H3N2)-like virus	A/Victoria/361/2011 (H3N2)- like virus	A/Texas/50/2012 (H3N2)	A/Hongkong/4801/2014 (H3N2)
**B**	B/Brisbane/60/2008-like virus	B/Brisbane/60/2008-like virus	B/Wisconsin/1/2010-like virus	B/Brisbane/60/2008 (Victoria)	B/Brisbane/60/2008 (Victoria)
**Percentage of circulating strain**	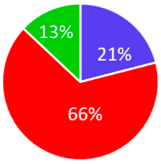	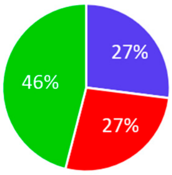	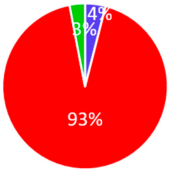	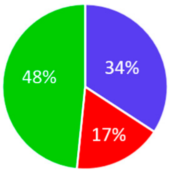	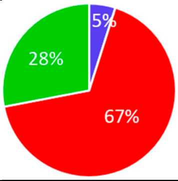

■ Influenza A (pdm09) ■ Influenza A (H3N2) ■ Influenza B

Abbreviations: A(H1N1), influenza A(H1N1)pdm09; A(H3N2), influenza A(H3N2); B, influenza B. **Notes:** Recommended Southern Hemisphere influenza vaccine components are shown for the years with available descriptive strain-context information (2011–2015). The B component shown for each year is the influenza B vaccine component recommended for that Southern Hemisphere season. For the present 2013–2024 study, only the overlapping study seasons were used to inform interpretation. These data are provided to support interpretation of year-to-year variation in vaccine effectiveness in the main analysis and should not be interpreted as a formal strain-match analysis across the full 2013–2024 study period or as patient-level vaccine formulation data.

Table S3. Circulating influenza strains by subtype/lineage and vaccine-strain correspondence in Thailand for years with available descriptive strain-context information, 2011–2015.

Year	Influenza Viruses		
	A (H1N1)	A (H3N2)	B
2011	A/California/7/2009 (H1N1)pdm09 - **100%**	A/Perth/16/2009 (H3N2) - **86.36%**; A/Victoria/361/2011 (H3N2) - 13.64%	B/Brisbane/60/2008 (Victoria) - **83.7%**; B/Florida/60/2008 (Yamagata) - 3.7%; B/Wisconsin/01/2010 (Yamagata) - 12.6%
2012	A/California/7/2009 (H1N1)pdm09 - **100%**	A/Perth/16/2009 (H3N2) - 0%; A/Victoria/361/2011 (H3N2) - 100%	B/Brisbane/60/2008 (Victoria) - **26.3%**; B/Wisconsin/01/2010 (Yamagata) - 45.4%; B/Massachusetts/2/2012 (Yamagata) - 28.3%
2013	A/California/7/2009 (H1N1)pdm09 - **100%**	A/Victoria/361/2011 (H3N2) or A/Texas/50/2012 (H3N2)* - **100%**	B/Wisconsin/01/2010 (Yamagata) - **15.4%**; B/Brisbane/60/2008 (Victoria) - 13.2%; B/Massachusetts/2/2012 (Yamagata) - 71.4%
2014	A/California/07/2009 (H1N1)pdm09 - **100%**	A/Texas/50/2012 (H3N2) - **50%**; A/Switzerland/9715293/2013 (H3N2) - 50%	B/Brisbane/60/2008 (Victoria) - **11.11%**; B/Massachusetts/02/2012 (Yamagata) - 62.22%; B/Phuket/3073/2013 (Yamagata) - 6.67%; B/Wisconsin/01/2010 (Yamagata) - 20.00%
2015	A/California/07/2009 (H1N1)pdm09 - **100%**	A/Hongkong/4801/2014 (H3N2) - **8.22%**	B/Brisbane/60/2008 (Victoria) - **9.38%**; B/Massachusetts/02/2012 (Yamagata) - 6.25%; B/Phuket/3073/2013 (Yamagata) - 84.38%

Abbreviations: A (H1N1), Influenza A(H1N1)pdm09 strains; A (H3N2), Influenza A(H3N2) strains; B, Influenza B strains. **Notes:** This table presents the proportions of circulating influenza strains by subtype and lineage in Thailand for the years with available descriptive strain-context information (2011–2015). The percentages shown in each cell represent the proportion of circulating strains identified within that subtype/lineage category for the corresponding year. Percentages shown in bold indicate circulating strains corresponding to the vaccine strain used in that year. * A/Texas/50/2012 is an A(H3N2) virus antigenically like the cell-propagated prototype virus A/Victoria/361/2011. For the present 2013–2024 study, only the overlapping study seasons were used to inform interpretation. These data are provided as contextual support for interpretation of year-to-year variation in vaccine effectiveness and should not be interpreted as a formal year-by-year strain-match analysis across the full 2013–2024 study period or as patient-level vaccine formulation data.
